# Discussion on *On the role of data, statistics and decisions in a pandemic* by Jahn et al (2022)

**DOI:** 10.1007/s10182-022-00450-y

**Published:** 2022-06-10

**Authors:** Ursula Berger, Göran Kauermann, Helmut Küchenhoff

**Affiliations:** Ludwig-Maximilians-University Munich, Munich, Germany

## Abstract

The authors make an important contribution presenting a comprehensive and thoughtful overview about the many different aspects of data, statistics and data analyses in times of the recent COVID-19 pandemic discussing all relevant topics. The paper certainly provides a very valuable reflection of what has been done, what could have been done and what needs to be done. We contribute here with a few comments and some additional issues. We do not discuss all chapters of Jahn et al. (AStA Adv Stat Anal, 2022. 10.1007/s10182-022-00439-7), but focus on those where our personal views and experiences might add some additional aspects.

## Decisions

Time is of the essence in pandemic response. Important political decisions, e.g. on contact restriction regulations or on border closures, must be made instantaneously to ensure their effectiveness. Therefore, a complex decision process as described by the authors in Chapter 5 was certainly not feasible in the first stages of the pandemic due to a general unpreparedness. Later in the pandemic, however, it is complex political structures (at least in Germany) that substantially hampered the development of a transparent and efficient process for making well-informed and scientifically sound decisions. In Germany, it was not until December 2021 that a national interdisciplinary expert panel was established, that is nearly more than two years after the first infections.^1^ Moreover, the obviously important scientific areas of epidemiology, statistics and public health were not sufficiently represented in this expert panel. Both these facts are to be criticized, above all as the German way is clearly in contrast to that of other countries. In UK the Royal Statistical Society built a COVID-19 task force, which was launched already in April 2020, with the purpose that the statistical society "can contribute its collective expertise to ... governments and public bodies, regarding statistical issues during the Covid-19 pandemic". This task force provided a forum where activities from statisticians were coordinated.^2^ In Germany, too, many statisticians and scientist focused their research on COVID-19 when the pandemic broke out. However, in the absence of a coordinating body much of the work remained unnoticed or had little impact in the political decision-making process. Thus, when setting up such a coordinating body, a relevant aspect is that it isn’t exclusionary to selected experts but that it is open to all research groups to contribute to ensure a high effectiveness. Such a coordinating body could forward requests from decision-makers or the media to experts and foster that evidence-based analyses receive appropriate attention.

## Reporting and communication

Scientific findings are of little value if they aren’t well communicated. At our university we founded the COVID-19 Data Analysis Group (CODAG@LMU) already in March 2020, when the first wave was hitting Germany. This group consists of statisticians, biometricians and epidemiologists who decided to devote their research to the analysis of available COVID-19 related data with the aim to provide fast solutions, stable models and valid forecasts so that decision makers in healthcare and politics could base their decisions on data. It was a novel role for us, for which we changed the way we disseminate our scientific results. As the pandemic demanded rapid response, we explicitly decided against the conventional way of dissemination scientific work via writing and submitting scientific articles to peer review journals. The extensive publishing process of scientific journals, justified in "normal times", simply could not have met the time demands of the pandemic, with new situations and new questions arising every week. For communication we chose instead a biweekly report of our newest statistical analyses on the current situation and on upcoming issues, the so-called CODAG^3^ Reports. This way of communication proved to be timely and showed reflection in media and with policy makers. However, in this way it was not possible to meet all criteria for scientific articles, mentioned by Jahn et al. (2022). To partly compensate for this shortcoming, we carefully and critically discussed each contribution and its conclusions among the group and always reported any limitations. While our approach can certainly not be recommended generally, it allowed us to contribute to a number of topics without the inevitable time delay of scientific peer-review publishing. We want to emphasize this explicitly, since we think that while the work by Jahn et al. (2022) lays out proper scientific and statistical management of getting from data to information, an effective dissemination of actual findings ensuring that they reach the decision makers in healthcare and politics in time, remains an open problem to be solved.

Most of the topics, which we discussed in our CODAG Reports, were important and timely questions related to the current course of the pandemic. This included the adequate and correct choice of surveyed parameters. We have argued frequently against the raw number of reported cases per 100 000 inhabitants, the so-called incidence, which still seems to be the most popular parameter in politics and media. First of all it is questionable, if the incidence is reflecting the burden of the pandemic, or if we should rather look at hospital or ICU admissions. Moreover, reported numbers are not robust against unreporting and are affected by reporting delays. The latter can be corrected by nowcasting, see (Schneble et al. 2021; De Nicola et al. 2022) or (Günther et al. 2021). For hospital admissions a cooperative approach on nowcasting has been initiated by Bracher et al. (2021). Here, different statistical approaches for estimating current hospital admissions for the German federal states contributed by different research groups are compared and the results are provided on a web-page with daily updates.^4^ The web-page includes an ensemble nowcast estimate and different tools for comparing models and checking previous nowcasts. These results find much notice and have also been used by some leading newspapers. However, it took months to convince policy makers that nowcasting techniques are required when analysing reported data.

## Statistical modelling

We appreciate the broad discussion of statistical models by Jahn et al. (2022) but would like to mention two further issues. Occasionally, natural experiments arise and can serve as valid data sources for causal inference. We refer to Berger et al. (2022) for an example. In that paper we were able to quantify the effect of mandatory tests at schools on the number of unreported, yet undetected infections. The natural experiment resulted since after the Easter vacation 2021 some Bavarian districts had school closure while others were open with mandatory tests. This built the framework of a natural experiment. Apparently, natural experiments can’t be planned, but a sensitivity to discover them is certainly helpful for statisticians and epidemiologists. We also want to mention that data fusion can be a valid approach if experimental or survey data are not available. We refer exemplary to Fritz and Kauermann (2022) where we combine data from Facebook, data from the Federal Statistical Office of Germany (DeStatis), incidence numbers and geospatial location in order to quantify the effect of the lockdown in 2020 as a non-pharmaceutical measure. Apparently, this approach is not free of drawbacks.

## Conclusion

We want to close by thanking and congratulating the authors for a very comprehensive and well elaborated report on how statistics can and should be used in pandemic times. We hope that this paper reaches readers beyond the statistical community.

## Footnotes

1 see https://www.bundesregierung.de/breg-de/bundesregierung/bundeskanzleramt/corona-expertinnenrat-der-bundesregierung.

2 see https://rss.org.uk/policy-campaigns/policy/covid-19-task-force/.

3 https://www.covid19.statistik.uni-muenchen.de/newsletter/index.html.

4 https://covid19nowcasthub.de/.

## References

[CR1] Berger, U., Fritz, C., Kauermann, G.: Reihentestungen an Schulen können die Dunkelziffer von COVID-19 Infektionen unter Schülern signifikant senken. Das Gesundheitswesen (to appear) (2022) 10.1055/a-1813-9778 PMC11248797 35675830

[CR2] Bracher J, Günther F, Heyder S, Hotz T, Küchenhoff H, Schienle M, Syliqi D, Weigert M, Wolffram D (2021). Eine kollaborative Plattform zur korrigierten Schätzung der Sieben-Tages Hospitalisierungsrate. Codag Bericht.

[CR3] De Nicola G, Schneble M, Kauermann G, Berger U (2022). Regional now- and forecasting for data reported with delay: towards surveillance of COVID-19 infections. AStA Adv. Stat. Anal..

[CR4] Fritz C, Kauermann G (2022). On the interplay of regional mobility, social connectedness, and the spread of covid-19 in germany. J. R. Stat. Soc. Ser. A R. Stat. Soc..

[CR5] Günther F, Bender A, Katz K, Küchenhoff H, Höhle M (2021). Nowcasting the COVID-19 pandemic in Bavaria. Biom. J..

[CR6] Jahn B, Friedrich S, Behnke J, Engel J, Garczarek U, Münnich R, Pauly M, Wilhelm A, Wolkenhauer O, Zwick M, Siebert U, Friede T (2022). On the role of data, statistics and decisions in a pandemic. AStA Adv. Stat. Anal..

[CR7] Schneble M, Nicola GD, Kauermann G, Berger U (2021). Nowcasting fatal COVID-19 Infections on a regional Level in Germany. Biom. J..

